# Lost in translation: What we have learned from attributes that do not translate from Arabidopsis to other plants

**DOI:** 10.1093/plcell/koaf036

**Published:** 2025-05-15

**Authors:** Adrienne H K Roeder, Andrew Bent, John T Lovell, John K McKay, Armando Bravo, Karina Medina-Jimenez, Kevin W Morimoto, Siobhán M Brady, Lei Hua, Julian M Hibberd, Silin Zhong, Francesca Cardinale, Ivan Visentin, Claudio Lovisolo, Matthew A Hannah, Alex A R Webb

**Affiliations:** Weill Institute for Cell and Molecular Biology and School of Integrative Plant Science, Section of Plant Biology, Cornell University, 239 Weill Hall, 526 Campus Rd., Ithaca, NY 14853, USA; Department of Plant Pathology, University of Wisconsin—Madison, Madison, WI 53706, USA; Genome Sequencing Center, HudsonAlpha Institute for Biotechnology, Huntsville, AL 35806, USA; US Department of Energy Joint Genome Institute, Berkeley, CA 94720, USA; Department of Soil and Crop Sciences, Colorado State University, Ft. Collins, CO 80523, USA; Donald Danforth Plant Science Center, St. Louis, MO 63132, USA; Donald Danforth Plant Science Center, St. Louis, MO 63132, USA; Howard Hughes Medical Institute, University of California, Davis, Davis, CA 95616, USA; Howard Hughes Medical Institute, University of California, Davis, Davis, CA 95616, USA; Department of Plant Sciences, University of Cambridge, Cambridge CB2 3EA, UK; Department of Plant Sciences, University of Cambridge, Cambridge CB2 3EA, UK; The State Key Laboratory of Agrobiotechnology, School of Life Sciences, The Chinese University of Hong Kong, Hong Kong, P.R. China; PlantStressLab, Department of Agricultural, Forest and Food Sciences, University of Turin, Grugliasco, TO 10095, Italy; PlantStressLab, Department of Agricultural, Forest and Food Sciences, University of Turin, Grugliasco, TO 10095, Italy; PlantStressLab, Department of Agricultural, Forest and Food Sciences, University of Turin, Grugliasco, TO 10095, Italy; BASF, BASF Belgium Coordination Center CommV, Technologiepark 101, 9052 Gent, Belgium; Department of Plant Sciences, University of Cambridge, Cambridge CB2 3EA, UK

## Abstract

Research in *Arabidopsis thaliana* has a powerful influence on our understanding of gene functions and pathways. However, not everything translates from Arabidopsis to crops and other plants. Here, a group of experts consider instances where translation has been lost and why such translation is not possible or is challenging. First, despite great efforts, floral dip transformation has not succeeded in other species outside Brassicaceae. Second, due to gene duplications and losses throughout evolution, it can be complex to establish which genes are orthologs of Arabidopsis genes. Third, during evolution Arabidopsis has lost arbuscular mycorrhizal symbiosis. Fourth, other plants have evolved specialized cell types that are not present in Arabidopsis. Fifth, similarly, C_4_ photosynthesis cannot be studied in Arabidopsis, which is a C_3_ plant. Sixth, many other plant species have larger genomes, which has given rise to innovations in transcriptional regulation that are not present in Arabidopsis. Seventh, phenotypes such as acclimation to water stress can be challenging to translate due to different measurement strategies. And eighth, while the circadian oscillator is conserved, there are important nuances in the roles of circadian regulators in crop plants. A key theme emerging across these vignettes is that even when translation is lost, insights can still be gained through comparison with Arabidopsis.

## Introduction

(Written by Adrienne Roeder, editor)

For the focus issue on Translational Research from Arabidopsis to Crop Plants and Beyond, we wanted to consider not only what does translate but also what does not translate. We view translation holistically as the gene functions, pathways, phenotypes, and technologies discovered and developed in Arabidopsis that inform our understanding of other plants. We invite our readers to look at the other reviews in this focus issue for a thorough discussion of findings that do translate from Arabidopsis to crops. Here we have asked experts to carefully consider what does not translate and why. These experts have written 8 vignettes about their areas of interest. One of the common themes that emerges from these articles is that loss or gain of a structure, function, cell type, or gene in evolution is a common reason why something does not translate from Arabidopsis to other plants. For example, loss of arbuscular mycorrhizal fungal symbiosis, loss and gain of genes with duplications, differences in the anatomy of the flower and the root, innovation of C_4_ photosynthesis, and novel transcriptional regulatory mechanisms in larger genomes are all barriers to translation. It is often challenging to publish well-designed but unsuccessful translation studies, but these results are valuable, and we encourage authors to share their negative results with the scientific community. Even in these systems that do not translate, another common theme emerging from the vignettes is that there is much still to learn from comparison with Arabidopsis. For example, studying gene loss in Arabidopsis helped to identify genes required for arbuscular mycorrhizal fungus symbiosis. Studying C_4_ photosynthesis genes in Arabidopsis has revealed their underlying functions that have then been coopted for C_4_ during evolution. This set of vignettes is by no means exhaustive and is instead meant to give a flavor as opposed to a complete picture. Please also see a companion review on challenges in translation to crop plants by Christobal Uauy et al. We hope you enjoy reading about what has been “lost in translation.”

## Floral dip transformation of Arabidopsis: how, and why not most other species?

(Written by Andrew Bent)

Twenty-six years ago it seemed too good to be true—or colossally unfair, for those working on other plant species—that of all plants, Arabidopsis could be stably transformed by simply dipping flowers in Agrobacterium and sucrose, collecting the progeny seeds and selecting transgenic T1 plants. Arabidopsis, with its small size and rapid generation time, hundreds of biologically interesting mutants, hundreds of scientists, millions in research grant support, and a full genome sequence on the way, was now transformable by simple floral dip (Clough and Bent 1998). The impact, already spreading from predecessor methods (Feldmann and Marks 1987; Bechtold et al. 1993; Chang et al. 1994), was substantial. Graduate students and postdocs routinely made and studied a few dozen transgenic lines to test their hypotheses within a few years. Gene discovery by map-based (positional) cloning was facilitated. Over 200,000 transgenic Arabidopsis lines with T-DNA insertions, saved as a random mutagenesis collection, became a turbocharged reverse genetics resource when DNA sequence adjacent to the T-DNA insertion was cataloged for each separate, saved line (Alonso et al. 2003; see also (Weigel et al. 2000). Researchers immediately tried to use floral dip transformation or related approaches in dozens of other plant species. And they have continued to try for 26 years, in dozens and dozens of efforts, some extensive and well-designed, but usually never published. For most researchers, floral transformation has not worked with the other species.

An exception: Floral dip transformation has worked quite well with other members of the Brassicaceae (e.g. Lu and Kang 2008; Xu et al. 2008; Chhikara et al. 2012; Hu et al. 2019). Beyond that, most reported successes have not been reproduced in other laboratories. A possibly encouraging sidebar story about reproducibility: the foundational work on nontissue culture transformation of Arabidopsis (Feldmann and Marks 1987), in which Agrobacterium was applied to Arabidopsis seeds, most often resulted in no transformants. But for the authors, it occasionally resulted in hundreds of solidly verified transformants. Other laboratories could not reproduce the method. Not all scientific findings are initially reproducible, even if they are true. A second, more discouraging sidebar probably explains many of the reported but nonreproducible reports of floral transformation success: Agrobacterium can persist on plants for weeks, or even generations, after its initial colonization (Cubero and Lopez 2005). This lends hope for future transformation attempts (see below) but also warns that use of PCR to verify transformation can unintentionally detect DNA from residual Agrobacterium that is interpreted as DNA stably integrated into a plant chromosome. Southern blots to detect T-DNA/plant-DNA junctions in restriction enzyme–digested plant DNA, T-DNA junction sequencing, and/or Mendelian segregation are among the standards required to rigorously demonstrate successful transformation.

**Figure 1.**
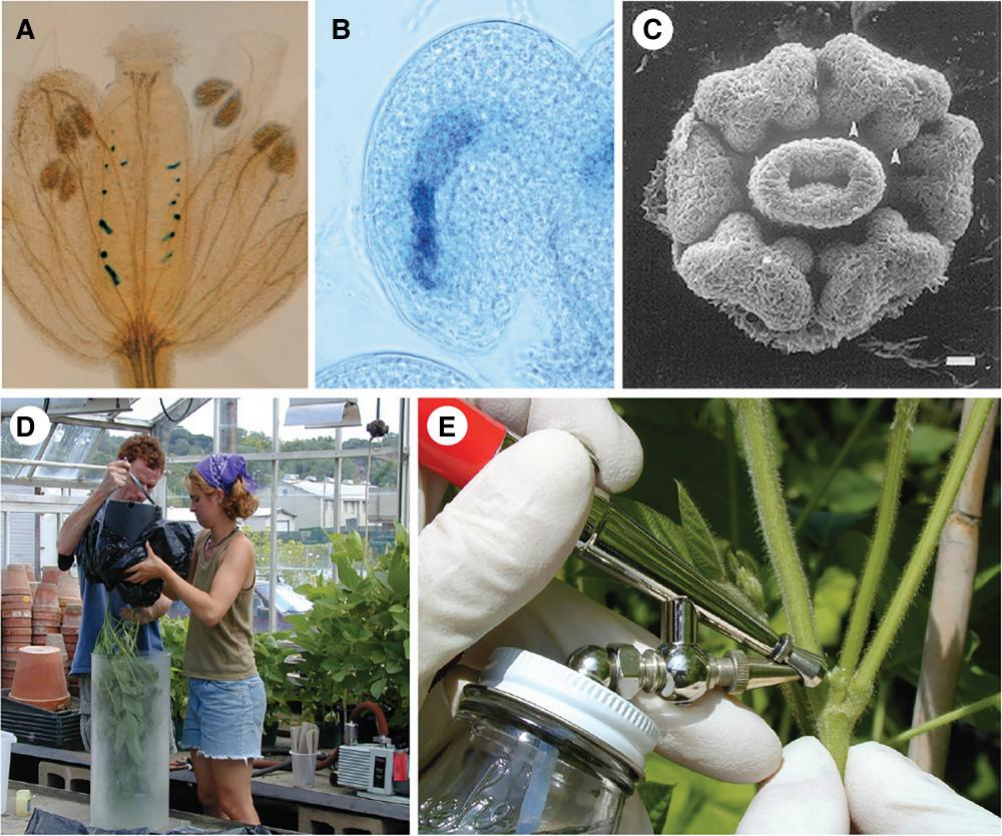
Floral dip transformation. GUS staining of **(A)** entire Arabidopsis flower, and **(B)** Arabidopsis ovule, after floral dip with Agrobacterium carrying intron-*GUS* in T-DNA. **C)** Immature Arabidopsis flower (gynoecium in center, surrounded by 6 anthers). Arabidopsis gynoecium (pistil) develops as an open vase; stigmatic cap only forms and closes locule 3 to 4 days before anthesis. **D)** Lost effort to translate floral dip to soybean (success rate 0/200,000 seeds). **E)** Possible air-brush method for applying Agrobacterium/sucrose/surfactant to floral primorida of soybean (or other plants). **A** and **B** are from (Desfeux et al. 2000), **C** is from (Bowman 1994).

The substantial pool of mechanistic information about Arabidopsis floral dip transformation seemingly could guide similar transformation of other species. In the original Feldmann and Marks work, individual transformants from the same T0 plant were shown to be independent transformation events, and transformants were always hemizygous (no homozygous or bi-allelic transformed progeny) (Feldmann and Marks 1987). The same was true in the seminal work of Bechtold et al., which launched accessible Arabidopsis transformation in laboratories around the world (Bechtold et al. 1993). Hemizygosity suggested gametophytes as the target for transformation after the divergence of the male and female germlines within flowers. Our laboratory and 2 other laboratories subsequently showed that female reproductive tissues are the primary target of floral dip transformation (Ye et al. 1999; Bechtold et al. 2000; Desfeux et al. 2000). This is crucially important. Externally applied Agrobacterium must reach the flower interior before locule closure so that it has access to gametophytes within developing ovules (Fig. 1). In Arabidopsis and other Brassica species, the gynoecium (pistil) develops as an open vase, with the stigmatic cap forming a closed locule only 3 to 4 days before anthesis (Bowman 1994). Hence vacuum infiltration and/or surfactant may be essential for any transformation success in plants from other taxonomic families just to get Agrobacterium into the deeper recesses of developing flowers. And in species where developing ovules become topologically isolated from the exterior much earlier, Agrobacterium apparently will have to be applied to proto-floral tissues correspondingly earlier. Or laboriously injected into individual flowers early enough, and with good aim for the locule. It also bears mention that many or most Arabidopsis flowers on a dipped plant are not transformed, while occasional “jackpot” flowers can contain many transformants (Fig. 1).

A handful of encouraging reports do exist of tissue culture-free floral transformation in non-Brassicaceae, for example, in *Setaria viridis* (Martins et al. 2015; Saha and Blumwald 2016; Van Eck 2018). A direct quote from a separate abstract, involving tomato floral bud transformation, is instructive:

“The imbibition of seeds with Agrobacterium suspension led to seed mortality. The vacuum infiltration of seedlings with Agrobacterium suspension led to sterility in surviving plants. Successful transformation could be achieved either by dipping of developing floral buds in the Agrobacterium suspension or by injecting Agrobacterium into the floral buds. Most floral buds subjected to dip as well as to injection either aborted or had arrested development. The pollination of surviving floral buds with pollen from wild-type plants yielded fruits bearing seeds. A transformation efficiency of 0.25% to 0.50% was obtained on floral dips/floral injections.” (Sharada et al. 2017)

The tomato method is not easy, but maybe it can work.

It bears note that, as of 26 years later, T-DNA and other sequence-indexed insertional mutagenesis collections have been developed for multiple other species (Ram et al. 2019; Nandety et al. 2023). RNAi and gene editing, high-quality reference genomes, public RNA-seq datasets, and other resources expedite progress in numerous plant species. Improved transformation methods are now available for many plant species (Altpeter et al. 2016). But for most plants, transformation is still a significant bottleneck requiring expert labor and significant expense (Altpeter et al. 2016). Our own work attempting floral dip transformation of soybean spanned 8 years and over 200,000 candidate seeds generated within greenhouses (Fig. 1). We varied soybean genotypes, *Agrobacterium* genotypes, flowering time, temperature, humidity, inoculation reagents, and application methods. We tested T-DNA transfer, plant defense responses, and other parameters. As in many other laboratories, we did not succeed. But the following observations can be summarized from our work and that of others.

What might contribute to future success in adapting Agrobacterium floral dip transformation methods to other species? First and foremost, attention to time of locule closure during flower development and associated innovations in Agrobacterium delivery (or persistence). Second, finding ways to minimize floral abortion and plant defense responses to the Agrobacterium (examples: Agrobacterium with a flagellin that does not elicit pattern-triggered immunity on that host species, effectors that suppress plant immune responses, use of chemicals that diminish immune responses, or plant accessions uniquely amenable to Agrobacterium) (Olhoft et al. 2003; De Saeger et al. 2021; Raman et al. 2022; Beritza et al. 2024). Third, enhancement of Agrobacterium T-DNA transfer and integration (Collier et al. 2018; De Saeger et al. 2021). Fourth, possible use of plant mutants, for example with incomplete locule closure (e.g. *CRABS-CLAW*/*crc* mutations; Desfeux et al. 2000), which could then be removed by outcrossing if necessary. Stacking of multiple incremental improvements may raise rare success rates up to a feasible frequency. There is hope. In the meantime, to paraphrase the movie Casablanca: We’ll always have Arabidopsis.

## Complexities of orthology when translating gene function from model species

(Written by John T. Lovell and John K. McKay)

Orthologs are defined as sets of genes across multiple species that share a common ancestor (Nichio et al. 2017; Emms and Kelly 2019). One-to-one orthologs are perhaps the single most powerful resource to translate information across genomes and species. Such genes likely encode proteins with similar activities and biological functions (Gabaldón and Koonin 2013); therefore, the putative effects of amino acid substitutions can be translated from genetic models like mice and rats to species that are less amenable to experimental manipulations, like humans or many domesticated animals. One-to-one orthologs are equally critical in plants, where functional annotations in *Arabidopsis thaliana*, rice, or other model species can be translated to crops that lack tools for functional genetics (Lye and Purugganan 2019). For example, in canola (*Brassica napus*), pod (silique) shattering was a major cause of yield losses in farmers’ fields. Academic and industry biologists were able to take advantage of the close evolutionary relationship of canola to Arabidopsis and use the extensive understanding of development and ease of manipulation to identify gene models controlling the development of siliques (Liljegren et al. 2000). Then via orthology, they identified the most similar gene models in canola and mapped existing polymorphism, and engineered new alleles to improve the crop by reducing pod shattering.

**Figure 2.**
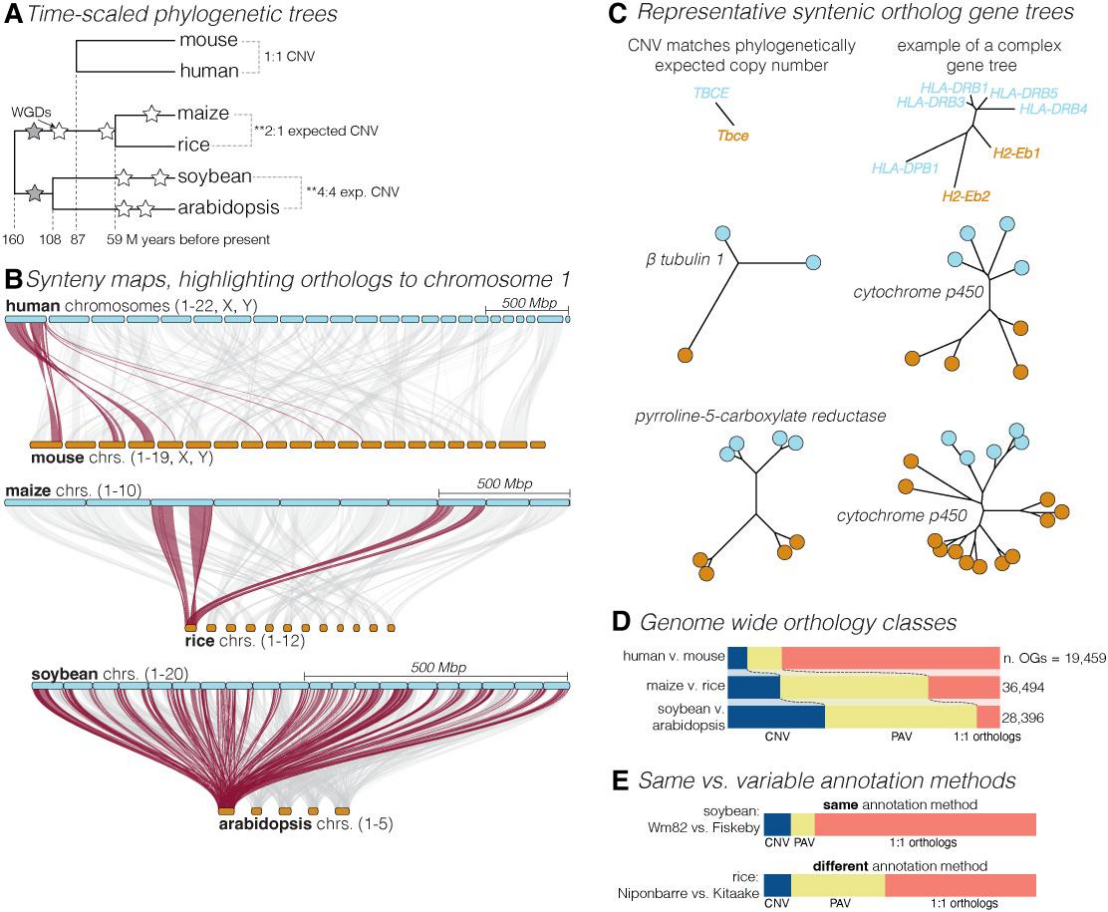
Comparison of expected copy number, synteny, and gene tree topology across 3 species. **A)** Time-calibrated phylogenies for the 3 contrasts. Each internal branch is dated by its median predicted divergence time. Stars indicate WGDs (white) and triplications (grey). **Indicates the expected copy number, based on ploidy increases following whole-genome duplications without consideration of subsequent diploidization. **B)** GENESPACE synteny maps between focal (blue; human, maize, soybean) and model (brown; mouse, rice, Arabidopsis) chromosomes. Synteny to the first chromosome of the focal species are colored red, while synteny to all other focal species’ chromosomes are light grey. Chromosome segments are scaled by their physical size (see independent key for each contrast for the length of a 500-Mb segment) and ordered by the alphanumeric chromosome ID order. **C)** Representative gene trees for sets of orthologs. For each contrast, 2 gene trees are presented. The left tree follows the expected copy number of orthologs in that contrast, given the whole-genome duplications presented in panel A. The right gene tree is a well-known complex gene tree with variable copy number. **D)** Tabulation of orthogroup classifications for the 3 contrasts: 1-to-1 orthologs are single copy in each genome (pink), PAV are missing in 1 genome (yellow), and CNV orthogroups (blue) are the remainder. **E)** Tabulation of orthogroup classifications for 2 pairs of genome annotations. Both annotations for the soybean contrast (top) were built by Phytozome with identical parameters and methods. The rice contrast (bottom) compares the genome annotation of “Nipponbarre,” which was originally annotated by Michigan State University (and is now hosted on NCBI), and the “Kitaake” cultivar, which was annotated by Phytozome. Colors and class orders follow panel D.

Despite our reliance on orthology for translational genomics across species, there are many evolutionary processes that lead to deviation from the expected 1-to-1 relationships among genes that arose from a single common ancestor (Panchy et al. 2016), including duplications in 1 or both lineages (copy number variation [CNV]), and loss via mutation to pseudogenes or deletions (presence-absence variation [PAV]) (McGrath and Lynch 2012; Panchy et al. 2016; Carretero-Paulet and Van de Peer 2020; Xiong et al. 2022). In the development of genetic and molecular evolution theory, it was largely assumed that gene duplication and redundancy were rare. Much of the early thinking was from a dosage perspective, when an organism is adapted to its environment, a doubling of any given gene product or protein is predicted to be deleterious (Spofford 1969; Ohno 1970). This assumption of uniformly 1:1 ortholog ratios matches biological observations in many systems, including mammals (e.g. human-mouse contrasts in Fig. 2), so theory on the fate of duplicated gene models is underdeveloped compared with allele frequency-based theory.

Despite origins in the 1960s (Spofford 1969; Ohno 1970), the idea that relaxed selection and subsequent evolution of new functions (neo-functionalization) following gene duplication has only recently been experimentally probed (Hittinger and Carroll 2007; McGrath and Lynch 2012). These studies have shown that multiple alleles of a recently duplicated gene model evolve by mutation, selection, and drift to have new functions (e.g. *sub1*; Bailey-Serres et al. 2010). In addition, there is an abundance of examples of duplication followed by loss of 1 copy, where under a model of both genetic drift as well as selection against a second copy, mutation eventually leads to pseudogenes.

When comparing sister species across relatively short evolutionary distances, we expect most gene models to show 1:1 orthology, in the absence of whole genome duplications (WGDs). Indeed, in mammals, there are strict 1:1 relationship between most orthologous genomic segments even across ∼100 m years of evolution (Fig. 1). As such, gene duplications can be ignored in many systems. However, plant lineages are riddled with recurrent WGDs followed by pseudogenization or neo-functionalization (Panchy et al. 2016), which has spurred evolutionary innovation across and within species. For example, WGDs are implicated as the major driver of diversification of all flowering plants (Soltis et al. 2009), grasses (McKain et al. 2016), and mustard crops (Walden et al. 2020), among others. Indeed, recent exploration of genomes and pan-genomes shows that gene duplication and loss is not an exception but instead the rule in flowering plants (e.g. Huang et al. 2021; Lovell et al. 2021) and is likely an understudied source of phenotypic evolution.

The complex evolutionary history of plant genomes makes translating functional information through 1-to-1 orthology not only difficult but also potentially uninformative. For example, there are 5 WGDs and 2 triplications between the last common ancestor of Arabidopsis and maize—if all genes were retained with their original function without gene loss, the only informative orthologs would have 24 copies in maize and 12 copies Arabidopsis. However, such sets of CNV orthologs (orthogroups) do not exist, and 96.3% of orthogroups have mean of ≤3 genes in each species. Indeed, both genomes have a reduced copy number back to what is thought to be nearly “normal” numbers of 25- to 40-k protein coding genes. Therefore, 100% of gene families that have diversified from a single common ancestor to maize and Arabidopsis have undergone some level of gene loss and potentially changes in gene function.

In plants, the role of gene loss and likely new functional evolution of paralogs is pervasive even at smaller phylogenetic scales. For example, maize and rice diverged ∼59 million years before present (m ybp; Fig. 2A), fewer years but likely similar number of generations to the common ancestor between human and mouse (∼96 m ybp). While 1:1 orthologs dominate between the 2 mammals (31,192 genes, 71.2%; Fig. 2D), between the 2 grasses, only 19,280 (24.7%) genes are 1:1 orthologs and the bulk show either PAV (31,042, 39.8%) or CNV (27,767, 35.6%). This pattern is even stronger between the more diverged (∼108 M ybp) contrast between the crop soybean and the closest genetic model species, Arabidopsis. In this case, only 4,824 (6.3%) of all genes are 1:1 orthologs (Fig. 2D).

In total, the nested whole-genome duplications can make even simple plant gene families as difficult to analyze as the most complex loci in mammals. For example, one of the most complex mammalian loci, HLA-DRB (Doxiadis et al. 2012), has 4 copies (plus HLA-DPB1) in the human genome and 2 in the mouse genome (Fig. 2C). A gene family like P5CR, which has kept all 4 copies expected from the 2 nested WGDs in both soybean and Arabidopsis, is equally (or more) complex than HLA-DRB (Fig. 2C). Those genes that, like HLA-DRB, originated with more than 1 copy display intractable gene tree topologies (e.g. Cytochrome P450, Fig. 2C) in plant lineages with nested WGDs.

The proximate causes of such variable gene content between plants is in part due to nested WGDs. Indeed, maize has incurred 1 WGD, both soybean and Arabidopsis have undergone 2 WGDs and both plant lineages diverged shortly after a WGD. However, WGDs and the subsequent genome reshuffling required to maintain reasonable chromosome numbers also makes it difficult to track genomic regions of interest (Fig. 2B) and thus contextualize the processes causing divergence from 1:1 orthology. While some lineages like grasses retain fairly stable gene order and chromosome number, genome structural reorganization in the ancestors of soybean and Arabidopsis causes large chromosomal segments to be related (syntenic) to every chromosome in the alternative species, requiring evolution-informed synteny tracking methods to determine orthologous candidate genes for genomic regions of interest (Lovell et al. 2022).

Genome annotation methodological differences compound the phylogenetic difficulties posed by plant comparative genomics. Mammalian genomes have remarkably conserved gene content, so homology and ab initio gene prediction serves these genomes well. However, gene content variation is wildly diverse in plants, and as such, to ensure proper annotation of true genes, evidence from RNA-seq, full-length cDNA, or other transcript sequencing is required (Abbas et al. 2024). However, the methods to integrate these resources contribute to abundance of gene PAV. For example, the polyploid soybean genome should have many more PAV gene families than the inbred diploid rice genome due to pseudogenization of redundant gene copies. However, we observe the opposite if we compare 2 rice genomes annotated with independent methods (including different sequencing resources and curators) with 2 soybean genomes annotated with identical input and methods: the odds of observing a PAV gene is 5.6× higher between the 2 rice genomes (*n* PAV = 14,331) as the 2 soybeans (*n* = 4,312; Fig. 2E).

Without selection to maintain function or evolve a new function, one of the duplicate paralogs will eventually become a pseudogene via mutation and drift (Lynch and Conery 2000). This poses another major challenge for theory, bioinformatics, and empirical studies: how can one define the point at which a gene alters or loses function along a continuum from perfect duplicates to diverged copies to pseudogenes? At the completion of this process, a pseudogene will lack the basic features of a coding gene and will not be translated into a protein; however, the exact point where the gene loses function is ambiguous and often cannot be unequivocally identified. It is similarly difficult to distinguish between the fates of pseudogenization vs neo- and subfunctionalization, a problem that is exacerbated by technical details. For example, there are multiple pipelines for annotating genomes that are more or less permissive of duplicated gene models that are approaching pseudogenization, like our rice and soybean examples (Fig. 2E). Using expression level as an indicator of retained function can be effective in some species, but in genomes that are mostly selfish elements (like many crops), much of the genome is expressed (Gault et al. 2018), further obscuring the breakpoint between retained and diverged function across duplicated sequences.

The bulk of population and comparative genomics analysis pipelines and methods, including pan-genome graphs, were developed by large, well-funded teams working on human, mouse, and related mammals. Approaches developed for vertebrates, which often require complete 1-to-1 syntenic and orthologous relationships, can be intractable when applied to the complex chromosomal and gene copy number evolution in most plant species. We now have the ability to assemble and annotate any given species at key evolutionary nodes. To leverage these resources to better understand gene function across plants, it will be critical to develop new evolutionary time-flexible algorithms that explicitly model gene loss, birth, and neo-functionalization following recurrent WGDs. There is also a need to further develop theory and testable models on the fate of duplicated genes that allows for the continuum from identical duplicate to diverged neo-functionalized or pseudogenized copy. In crops, there are many case studies of important traits that are caused by varying degrees of genetic redundancy, but a systematic approach is lacking. Explicit information on copy number variation and the degree of pseudogenization are not represented in the polymorphism matrices used in genotype to phenotype studies. Representing this variation and developing new genome-wide scans to associate these features with phenotypic variance will require new funding opportunities and coordination within the research community. Combined, such data and theory will then allow new genotype to phenotype models that can partition out the degree of phenotypic variance due to gene redundancy vs allelic variation.

### Methods

Protein-coding gene models were downloaded from NCBI (human, mouse, Niponbarre rice, Arabidopsis) or phytozome (soybean, maize, kitaake rice). Code for all analysis and URLs for the raw annotation files can be found on the github repository: https://github.com/jtlovell/lostInTranslation/tree/main. All analyses were performed in R v4.4.0 (R Core Team 2020). GENESPACE v1.3.1 (Lovell et al. 2022) with default settings was used to build syntenic and genome-wide phylogenetically hierarchical orthologs (via OrthoFinder v2.5.4; Emms and Kelly 2019) and to generate riparian plots. Downstream parsing and summarization was performed with data.table v.1.16.4 (Barrett et al. 2024). Gene trees were built with fasttree v2.1.11 (Price et al. 2010) from mafft v7.520 (Katoh and Standley 2013) peptide alignments, both with default settings. Gene trees were visualized with ape v5.8 (Paradis and Schliep 2019). The phylogenetic tree was built via timetree (https://timetree.org; Kumar et al. 2022).

## Loss of the widespread arbuscular mycorrhizal symbiosis

(Written by Armando Bravo and Karina Medina-Jimenez)

Land plants rely on symbiotic relationships with other organisms to survive and thrive. Among these mutually beneficial interactions, arbuscular mycorrhizal (AM) symbiosis is the most widespread and ancient. In exchange for carbon from the plant, AM fungi facilitate the critical uptake of mineral nutrients by plants, particularly in nutrient-poor soils, carrying profound ecological and evolutionary implications for all terrestrial life (Smith and Read 2008).

The relationship between soil-living AM fungi and plants originated at the dawn of land plant evolution and has been maintained in most plant lineages, from rootless bryophytes to flowering plants, including most modern crops. However, around 20% of plant species have lost their ability to engage in functional mutualism with AM fungi (Wang and Qiu 2006). This loss of AM symbiosis has occurred multiple times throughout evolution across various lineages. Nonhost plants include those with specialized nutritional strategies, such as carnivorous or parasitic plants, and those that thrive in conditions challenging for AM fungi, like aquatic, arid, or saline environments (Cosme et al. 2018). Even more fascinating are plants that have adapted to the loss of AM symbiosis for reasons that are not entirely clear, like *Arabidopsis*.

**Figure 3.**
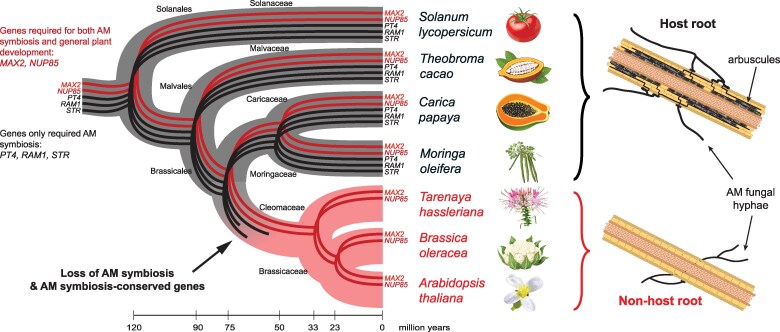
Loss of arbuscular mycorrhizal (AM) symbiosis in *Arabidopsis*. Phylogenetic tree of selected flowering plant species that shows the loss of genes required to engage in AM symbiosis in some plants from the Brassicales order, including *Arabidopsis thaliana*. Red represents the clades that lost the ability for AM symbiosis. Examples of known genes are included in the tree, showing how some genes are lost during evolution. Black genes represent genes conserved for AM symbiosis, and red genes are those with functions in both AM symbiosis and other important developmental pathways. A rough time scale is included to provide context and is based on (Hohmann et al. 2015). Diagrams represent root segments that successfully (upper) or unsuccessfully (lower) engage in symbiosis with AM fungi.

*Arabidopsis thaliana* is not a host for AM fungi. Like all members of the Brassicaceae family, this species lost the ability to engage in AM symbiosis due to the lack of necessary genes for this mutualism (Delaux et al. 2014; Cosme et al. 2018) (Fig. 3). Although Brassicaceae, like many other nonhost plants, can be colonized by AM fungi, arbuscules—the fungal structures formed inside cells for nutrient exchange—do not form, making the symbiosis nonfunctional (Fernández et al. 2019). Importantly, *Arabidopsis* alone cannot sustain the growth of obligate biotrophic AM fungi without neighboring true host plants to support the fungi, which is required for this mutualism (Veiga et al. 2013; Trautwig et al. 2023).

The production of plant glucosinolates, anti-microbial secondary metabolites, has been suggested as a possible cause for this loss (Sharma et al. 2023). However, evidence supporting this hypothesis is conflicting, especially regarding the impact of these chemicals on AM fungal growth (Glenn et al. 1988; Anthony et al. 2020). Notably, AM-host plants from the order Brassicales, such as papaya and *Moringa oleifera*, also produce glucosinolates and can still engage in AM symbiosis (Lopez-Rodriguez et al. 2020; Sharma et al. 2023). An alternate hypothesis is that *Arabidopsis* lost AM symbiosis due to beneficial interactions with other organisms, like fungal endophytes (Hiruma et al. 2016; Almario et al. 2022). These fungi can promote plant growth and phosphorus uptake, but the examples thus far are rarely found associated with natural populations of *Arabidopsis thaliana*. Additionally, no reciprocal exchange of carbon has been demonstrated for these interactions, unlike the well-characterized AM symbiosis. Nevertheless, *Arabidopsis* serves as a prime example of how plants have evolved alternative strategies for nutrition and the management of beneficial symbioses (Voges et al. 2019).

Overall, the loss of AM symbiosis in *Arabidopsis* and Brassicaceae highlights the complex evolutionary adaptations plants undergo, potentially favoring other beneficial symbiotic relationships over traditional mycorrhizal associations. However, due to this loss, *Arabidopsis* is unsuitable as a model for AM symbiosis research. Scientists must consider alternative plant species, despite the availability of genetic tools, established populations, and decades of research in *Arabidopsis* genetics.

### What can we learn about AM symbiosis by studying *Arabidopsis*?

Despite losing the ability to engage in functional symbiosis with AM fungi, there are many lessons we can learn from *Arabidopsis* about this important mutualistic association. When the first few genes required for AM symbiosis were identified in flowering plants, many were found to be missing from *Arabidopsis*, the only nonhost plant for AM fungi with a sequenced genome at the time. This led to the hypothesis that some genes have been selected and conserved through evolution solely for their function in AM symbiosis. In the following years, advances in DNA sequencing and more sequenced plant genomes made it possible to compare multiple genomes and identify genes uniquely present in plants that can engage in AM symbiosis. This was successfully done through comparative phylogenomics, a powerful approach based on evolutionary hypotheses that identified a set of genes conserved only in AM-host plants (Delaux et al. 2014; Favre et al. 2014; Bravo et al. 2016). This set of genes does not include all the genes required for successful symbiosis, as many are still present in nonhost plants, including *Arabidopsis*. Gene retention in nonhost plants is hypothesized to be due to additional gene functions and selection pressure. Significant work on these nonsymbiotic functions has led to functional characterization in *Arabidopsis*, aiding in the understanding of AM symbiosis. Examples include components of the nuclear pore complex, required for the initial perception of the fungus, and signaling through the hormone strigolactone, which activates AM fungal metabolism (Kohlen et al. 2011; Parry 2014; Rozpądek et al. 2018).

Symbiotic genes conserved exclusively in AM-host plants have roles and molecular functions that remain largely unexplored. While many of these genes encode proteins with broad, generic functions, such as transcription factors, transporters, and enzymes, their specific contributions to AM symbiosis remain unclear. In contrast to genes found in both host and non-host plants, many symbiotic genes exclusive for AM-host plants are predominantly active during symbiosis, specifically in the root cortex cells that host the fungus (MacLean et al. 2017; Hornstein and Sederoff 2024). Studies have shown that many of these genes are critical for fine-tuning or modifying broader pathways to support mutualism with AM fungi, achieving this innovation without the need to “reinvent the wheel”. Interestingly, some of these genes are also conserved in nonflowering plants, suggesting they represent core functions essential for AM symbiosis in all land plants (Radhakrishnan et al. 2020; Sgroi et al. 2024).

By studying the gene loss in *Arabidopsis* and other nonhost plants, we not only gain insights into the specific genetic components required for AM symbiosis but also enhance our understanding of the broader process of gene loss and its evolutionary implications. This research sheds light on how plants adapt to different ecological niches and the evolutionary pressures that shape their genomes.

Based on progress in the field and the advances in synthetic biology, we can further expand our understanding of AM symbiosis by asking the question “Can we restore symbiosis in a nonhost?” Answering this question will define the basic genes that make a mutualistic interaction work, how they are regulated, and how they interact with each other. We can investigate these questions while also tinkering with the genes to try to restore the capacity of plants like *Arabidopsis* to successfully engage in a symbiotic association with AM fungi (Hornstein and Sederoff 2024). This research has the potential to further expand the already profound impact that AM symbiosis has on plants and agriculture, creating more resilient and sustainable agricultural systems. It could also turn *Arabidopsis* into a model for AM symbiosis research.

## Different forms, new functions–the successes and limitations of Arabidopsis root anatomy

(Written by Kevin W. Morimoto, Siobhán M. Brady)

Research on the model species *Arabidopsis thaliana* has revolutionized our understanding of plant biology. With its well-annotated small genome, transformation capability, and short generation time, a vast number of resources have been developed for this species allowing deep insight into its development. For example, genome-scale mutant screens using Arabidopsis insertion mutant resources have uncovered critical regulators governing cell type development. The advent and improvement of cell type and cell-specific methodologies to map gene expression at unprecedented spatiotemporal resolution across Arabidopsis plant development as well as its dynamic responses to the environment (Brady et al. 2007; Lopez-Anido et al. 2021; Shahan et al. 2022) has allowed greater resolution into regulation and function of specific tissues. In turn, these resources have enabled studies elucidating cell type form and function.

A fundamental assumption underlying the use of a model species is that findings in that species are generalizable to other relatives. In some cases, this is indeed true. For example, recent work suggests conservation of function in regulation of Casparian Strip formation by *MYB36* in the root endodermis in Arabidopsis, tomato, and rice (Wang et al. 2022a, 2022b; Manzano et al. 2025). Much of the knowledge about molecular mechanisms governing root xylem differentiation also come from experiments in Arabidopsis; studies examining the transcription factors *SlCORONA-LIKE1* (*SlCNAL1*), *SlPHABULOSA/PHAVOLUTA-LIKE1* (*SlPHB/PHV-LIKE1*), and *SlVASCULAR-RELATED NAC-DOMAIN6* (*SlVND6*) have shown conservation in gene function between Arabidopsis and tomato for xylem cell specification and differentiation (Kajala et al. 2021). Other well-known examples of fundamental studies in Arabidopsis that demonstrated translational applications in other species are those that explored floral development. Studies on the *AtAPETALA1*, *AtAPETALA2*, *AtAPETALA3*, *AtAGAMOUS*, and *AtPISTILLATA* genes led to the development of the ABCE model governing floral organ identity (Bowman and Moyroud 2024). This model has since been used as a basis to identify floral patterning genes in other species such as tomato and petunia as well as to determine how variation in these genes leads to floral patterning diversity in species such as tulip, where variation in B-class gene expression leads to tulip tepal formation (Kanno et al. 2003; Monniaux and Vandenbussche 2023).

While these studies in Arabidopsis have been fundamental to understanding the underlying biology in other plant species, the direct translation of these findings becomes limited when considering species with anatomical traits not present in Arabidopsis. Notably, the root anatomy of multiple crop species starkly demonstrates this deviation from the conventional Arabidopsis root form and will be the focus of the following examples presented here. Arabidopsis root anatomy consists of concentrically layered cell types, with the bilaterally patterned vascular tissues (xylem, phloem and procambium) surrounded by the radially symmetrical pericycle, endodermis, single cortex layer, and finally the epidermis as the outermost cell layer (Dolan et al. 1993). Contrastingly, the roots of other species, while also possessing similar radial anatomy, contain multiple cortical layers. Tomato, rice, and maize contain an average of 3, 7 to 12, and 6 to 16 cortex cell layers, respectively (Ron et al. 2013; Chimungu et al. 2014; Zhang et al. 2021). Such a large diversity in cortex cell file number raises numerous questions about the potential functions of a multilayered cortex and which genes regulate cortex specification and layer number. *AtSHORTROOT* (*AtSHR*) in Arabidopsis regulates the initial cell asymmetric division that produces the endodermis and single cortex layer (Helariutta et al. 2000). Further studies in maize, rice, and setaria have shown that their *SHR* genes are involved in the production of a multiseriate (multilayer) cortex (Henry et al. 2017; Ortiz-Ramrez et al. 2021).

Functional specialization of individual layers within the multiseriate cortex enables interaction with the external environment. Thus, determination of the molecular mechanisms that produce a multiseriate cortex are important to both understand and breed plants that are better able to interact with a changing environment. For example, as described in the vignette by A. Bravo and K. Medina-Jimenez, it is known that mutualistic symbioses with arbuscular mycorrhizal fungi (AM fungi) occur through the root cortex (Choi et al. 2018). Research on maize positively associates the colonization of AM fungi with increased cortex cell file number, suggesting that more cortex layers could form the basis of increased fungal colonization (Galindo-Castañeda et al. 2019). However, with only 1 cortex layer and its inability to undergo AM symbiosis (Veiga et al. 2013), research in Arabidopsis is limited in translation to other species.

**Figure 4.**
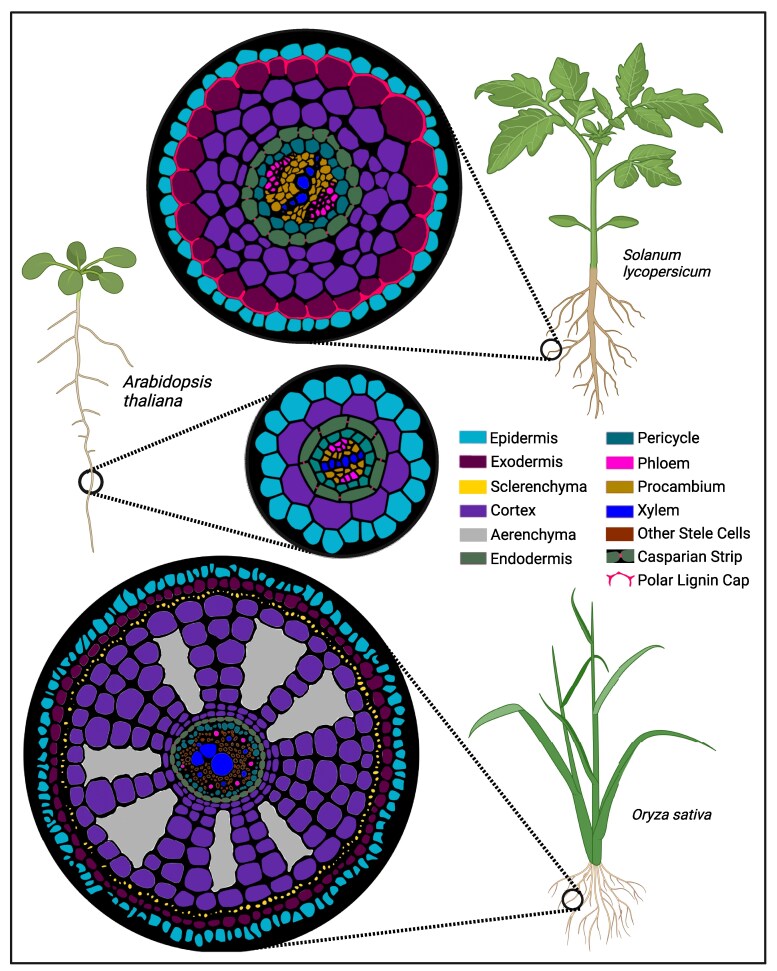
Schematic of tomato, Arabidopsis, and rice plants with cross-sectional diagrams depicting their respective root anatomies highlighting the polar lignin cap in *Solanum lycopersicum* (tomato) and aerenchyma in *Oryza sativa* (rice). Root legend cell type colors are listed in order from the outermost layer to the innermost layer as in *Oryza sativa*. Partially created with BioRender.com. Rice root image is adapted from BAR (bar.utoronto.ca) and Kajala et al. 2021.

Groups of cortex cells are also known to undergo cell death to form empty regions called aerenchyma. Aerenchyma are absent in Arabidopsis but comprise a large portion of root cross-sectional area in many species including rice and maize (Fig. 4). Several studies have shown these structures to be advantageous to growth in hypoxic conditions in rice by facilitating the movement of oxygen throughout the root as well as drought tolerance in maize (Dalle Carbonare et al. 2023; Schneider et al. 2023). Other studies have suggested aerenchyma as a resistance mechanism against pathogens, with one finding that *Fusarium verticillioides* infection and maize aerenchyma are inversely associated (Galindo-Castañeda et al. 2019). With the exception of the identification of *ZmbHLH121* in maize that controls aerenchyma formation in a specific root type, the genes that control aerenchyma are unknown (Schneider et al. 2023). As such, these 2 examples of cortex layer differences between Arabidopsis and other plant species, as well as the paucity of genes identified to control their development, highlight the limitations of translating research from Arabidopsis to other species.

Another notable anatomical distinction between Arabidopsis and other species is the presence of a root exodermis within the multiseriate cortex. The exodermis is estimated to be present in over 90% of angiosperms and is characterized by both its location directly beneath the epidermis and the deposition of cell wall polymers lignin and/or suberin (Perumalla et al. 1990; Liu and Kreszies 2023). The exodermis of crop species such as tomato, as well as other Solanaceous relatives, deposits a polar lignin cap on the epidermis face and anticlinal walls of each exodermis cell (Manzano et al. 2025 ) (Fig. 4). Cell type resolution-aided targeted mutant analysis of several known genes required for endodermis lignification in Arabidopsis were tested for function in tomato exodermis lignification; however, there were no resulting phenotypes, indicating a regulatory mechanism distinct from that of the endodermis (Manzano et al. 2025). Here, the translational capacity of Arabidopsis to other species is clear: known root endodermis lignification regulators in Arabidopsis do not appear to regulate exodermis lignification. So far, no activators of exodermis lignification have been identified. While the polar lignin cap functions as an apoplastic barrier similarly to the Casparian Strip, its distinct morphology suggests additional functions distinct from the Casparian Strip (Manzano et al. 2025). However, without the genetic resources to directly manipulate the polar lignin cap, such functions remain unknown.

These examples of root anatomical differences between Arabidopsis and tomato, rice, and maize serve to highlight the limitations of direct translation between research in Arabidopsis and other plant species. While the development of powerful genetic tools in Arabidopsis has fueled groundbreaking discoveries in plant cell type function, a larger effort toward the development of these tools in other species with different anatomical traits is warranted. For example, the development of cell type or cellular resolution spatial transcriptomic atlases of species with unique anatomical features distinct from Arabidopsis, as well as genetic (mutant) resources would aid in discovery of novel gene and cell type function. Indeed, recent multi-laboratory collaborations have yielded such resources at cell type resolution for this in agronomically important species such as rice, maize, and tomato (Kajala et al. 2021; Xu et al. 2021; Reynoso et al. 2022; Cantó-Pastor et al. 2024). Such resources may also serve as powerful tools for research in other closely related species for which translation from Arabidopsis is also limited due to differences in development and anatomy. Improvement in the translational potential of research findings will be bolstered through the continued development of such research in species outside of Arabidopsis.

## From C_3_ Arabidopsis to C_4_ photosynthesis

(Written by Lei Hua and Julian M. Hibberd)

**Figure 5.**
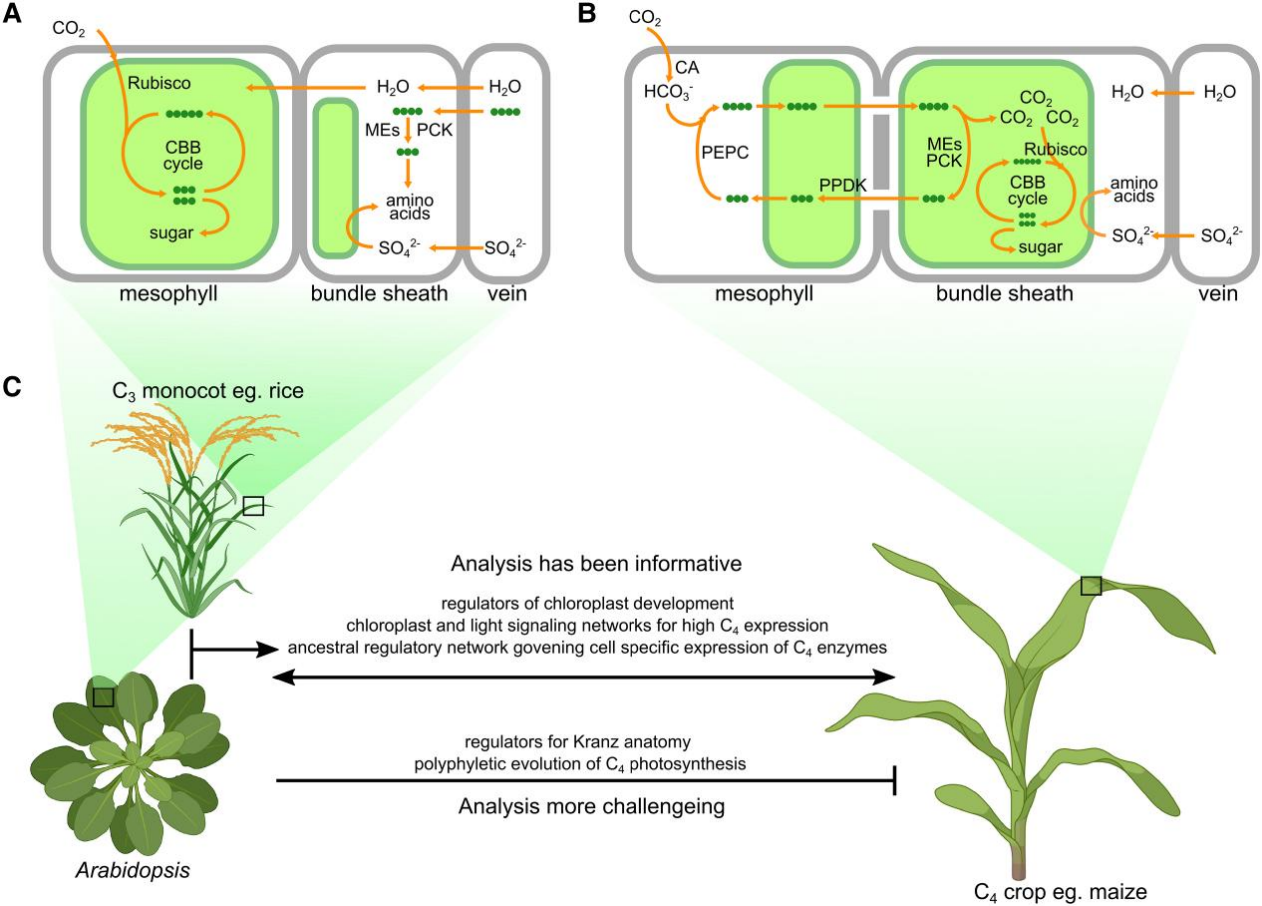
Molecular networks conserved across species aid translation from C_3_ to C_4_ photosynthesis. **A)** In C_3_ plants, assimilation of CO_2_ primarily occurs in mesophyll cells, with the bundle sheath also contributing to sulfur assimilation, water transport, and decarboxylation of C_4_ acids derived from the transpiration stream. **B)** C_4_ photosynthesis involves the coordinated function of mesophyll and bundle sheath cells to enhance carbon fixation efficiency. Carbon atoms are indicated by dots. **C)** Summary of areas where analysis of Arabidopsis has been informative, as well as areas where predictions are more challenging.

The photosynthetic process is conserved in most plants. One striking exception relates to the mechanism allowing carbon fixation that varies depending on whether the initial product contains 3 or 4 carbons. Species have therefore been categorized as using C_3_ or C_4_ photosynthesis and Crassulacean acid metabolism (Sage 2004). C_3_ photosynthesis (Fig. 5A) is considered ancestral, but from ∼30 million years ago in response to reductions in atmospheric CO_2_ and thus higher rates of photorespiration, the C_4_ pathway evolved in over 60 lineages (Christin et al. 2008, 2011; Sage et al. 2012).

In a few species the C_4_ pathway operates in large single cells (Voznesenskaya et al. 2001; Jurić et al. 2017), but in most CO_2_ is concentrated in specific cells such as the bundle sheath arranged in concentric circles around veins to generate Kranz anatomy (Langdale 2011). CO_2_ is initially converted to HCO_3_^−^ by carbonic anhydrase (CA) and then fixed into a C_4_ acid by phospho*enol*pyruvate carboxylase (PEPC) in mesophyll cells. C_4_ acids then diffuse into bundle sheath cells where decarboxylation releases high concentrations of CO_2_ around Rubisco. Decarboxylation is facilitated by 1 or more enzymes, NADP malic enzyme (NADP-ME), NAD malic enzyme (NAD-ME), or phospho*enol*pyruvate carboxykinase (PEP-CK) (Furbank 2011). To complete the cycle, the CO_2_ acceptor phosphoenolpyruvate (PEP) is regenerated by pyruvate orthophosphate dikinase (PPDK) in mesophyll cells (Fig. 5, A and B).

Although Arabidopsis does not use C_4_ photosynthesis or possess Kranz anatomy, it has been useful in understanding many traits underpinning the complex C_4_ phenotype. Much of the genetic basis of photomorphogenesis was elucidated with Arabidopsis (Nemhauser and Chory 2002), and the same was true for sucrose and starch biosynthesis (Stitt et al. 2010), as well as leaf and chloroplast development (Cackett et al. 2022; Lv et al. 2023). All these processes are critical for both C_3_ and C_4_ photosynthesis. Moreover, genes allowing photorespiration were identified in Arabidopsis (Somerville and Ogren 1979, 1980; Somerville 2001), and it played a key role in our understanding of the ancestral systems in C_3_ leaves that have been modified during evolution of the C_4_ pathway. For example, Arabidopsis leaves contain bundle sheath cells that are important for sulfur assimilation, hydraulic maintenance, and response to high light (Fryer et al. 2002; Leegood 2008; Griffiths et al. 2013; Sade et al. 2014; Xiong et al. 2021). These cells contain functional chloroplasts (Kinsman and Pyke 1998; Fryer et al. 2002) and silencing chlorophyll biosynthesis in these cells showed that their photosynthetic activity impacts on plant growth and fitness (Janacek et al. 2009). Moreover, genetic studies in Arabidopsis found that CA proteins regulate stomatal responses to CO_2_ (Hu et al. 2010), and PEPC plays a crucial role in modulating carbon and nitrogen metabolism (Shi et al. 2015). NADP-ME assists in the oxidative pentose phosphate pathway and fatty acid metabolism (Wheeler et al. 2005) and defense against pathogens (Voll et al. 2012). NAD-ME plays important roles in coordinating nitrogen and carbon metabolism by providing pyruvate to the tricarboxylic acid cycle cycle (Tronconi et al. 2008). There is also evidence that proteins of the C_4_ cycle act cooperatively in the C_3_ state. For example, PPDK and PEPCK allow gluconeogenesis during seedling establishment (Rylott et al. 2003; Eastmond et al. 2015), and both are implicated in nitrogen remobilization during leaf senescence (Lin and Wu 2004; Taylor et al. 2010). Midveins of Arabidopsis leaves possess high activities of C_4_ acid decarboxylases to release CO_2_ from organic acids from the xylem stream for photosynthesis (Brown et al. 2010). This trait is observed not only in other dicotyledons (Hibberd and Quick 2002) but also in rice (Shen et al. 2016). In fact, transcripts encoding these proteins are preferentially expressed in the bundle sheath of Arabidopsis and rice (Aubry et al. 2014; Hua et al. 2021). Deep sequencing of transcripts associated with Arabidopsis bundle sheath ribosomes highlighted roles in sulfur metabolism and glucosinolate biosynthesis (Aubry et al. 2014), and analysis of rice revealed conserved patterns of gene expression in sulfur assimilation, water transport, and jasmonic acid biosynthesis (Hua et al. 2021). Together, these data imply that the C_4_ bundle sheath has developed from a cell-type allowing photosynthesis with elements of the C_4_ pathway but in particular specialized in sulfur assimilation, water transport, and jasmonic acid biosynthesis (Fig. 5, A and B).

Arabidopsis has informed our understanding of regulatory changes required for C_4_ photosynthesis (Fig. 5C). For example, duons responsible for bundle sheath–specific expression in C_4_  *Gynandropsis gynandra*, as well as motifs for mesophyll specific expression, are found in Arabidopsis orthologs (Williams et al. 2016; Reyna-Llorens et al. 2018). As these motifs do not produce cell-specific expression in Arabidopsis, this indicates alternations in *trans* are required for C_4_ photosynthesis. Expression of C_4_ genes in C_3_ is lower than in C_4_ plants (Brautigam et al. 2011; Furbank et al. 2023). Comparative analysis of Arabidopsis and C_4_  *G. gynandra* showed that increased response of C_4_ genes to light in the C_4_ state is associated with gain of light-responsive elements (Singh et al. 2023). C_4_ orthologs are co-regulated with photosynthesis genes and controlled by both light and chloroplast-to-nucleus signaling in Arabidopsis, but in C_4_ leaves this regulation becomes increasingly dependent on the chloroplast (Burgess et al. 2016). Taken together, Arabidopsis shows that C_4_ photosynthesis has co-opted pre-existing gene regulatory networks from C_3_ species.

Despite insights from Arabidopsis into processes associated with C_4_ photosynthesis, there are limitations (Fig. 5C). Specifically, fundamental differences in leaf development between dicotyledons and monocotyledons mean work on Arabidopsis has not predicted regulators of Kranz anatomy (Sedelnikova et al. 2018). For example, although SCARECROW (SCR) is implicated in bundle sheath development in Arabidopsis (Cui et al. 2014), in rice it regulates stomatal development (Hughes and Langdale 2022), in maize it drives mesophyll proliferation (Hughes et al. 2019; Hughes and Langdale 2022), and in Setaria it manages both processes (Hughes et al. 2023). More generally, most lineages that evolved C_4_ photosynthesis are distantly related to Arabidopsis, and gene copies and function may have diverged and the *cis* and *trans*-code affecting gene regulation may be distinct. At least 5 WGDs separate dicotyledons and monocotyledons (Lee et al. 2013) allowing rearrangements, gene gains, and losses to reshape regulatory networks (Spannagl et al. 2011). In fact, more than 20% of Arabidopsis transcription factors downstream of photoreceptors are absent in grasses (Wang et al. 2017), and only ∼13% of Arabidopsis bundle sheath preferential genes were also expressed in these cells in rice (Hua et al. 2021). So far, bundle sheath– or mesophyll-specific promoters that work effectively in C_4_ crops have not been identified in Arabidopsis. That said, deeply conserved regulators do exist. The GOLDEN2-LIKE (GLK) transcription factors, first characterized in maize (Hall et al. 1998; Rossini et al. 2001), act as master regulators of chloroplast development in a broad range of species (Fitter et al. 2002; Tu et al. 2022; Hernández-Verdeja and Lundgren 2024) and play a crucial role in the development of bundle sheath chloroplasts in other C_4_ grasses (Lambret-Frotte et al. 2024). Similarly, GATA transcription factors GATA NITRATE-INDUCIBLE CARBON-METABOLISM-INVOLVED (GNC) and CYTOKININ-RESPONSIVE GATA1 (CGA1) maintain conserved roles in chloroplast biogenesis in both Arabidopsis and rice (Hudson et al. 2013; Bastakis et al. 2018; Zubo et al. 2018; Lee et al. 2021). An enhancer of bundle sheath gene expression from rice is able to specify the same patterning in Arabidopsis (Hua et al. 2024), and photosynthesis genes from C_4_ sorghum appear to have recruited DNA-binding with One Finger (DOF) elements that control bundle sheath expression in C_3_ rice (Swift et al. 2024). It is therefore possible that identifying and utilizing consensus molecular networks through comparative analysis with monocotyledons such as maize or rice will generate a more predictive framework for application to both C_3_ and C_4_ crops (Fig. 5C).

In summary, while Arabidopsis serves as a powerful model to understand fundamental biochemical and regulatory pathways underlying photosynthesis, it is unlikely to provide full insight into a trait that is found in over 60 independent lineages of angiosperms. Translating knowledge from Arabidopsis to C_4_ species will need careful consideration of phylogenetic distance and the use of closely related C_3_ species to the C_4_ plants being studied.

## Larger genome gave rise to transcriptional regulatory innovation

(Written by Silin Zhong)

**Figure 6.**
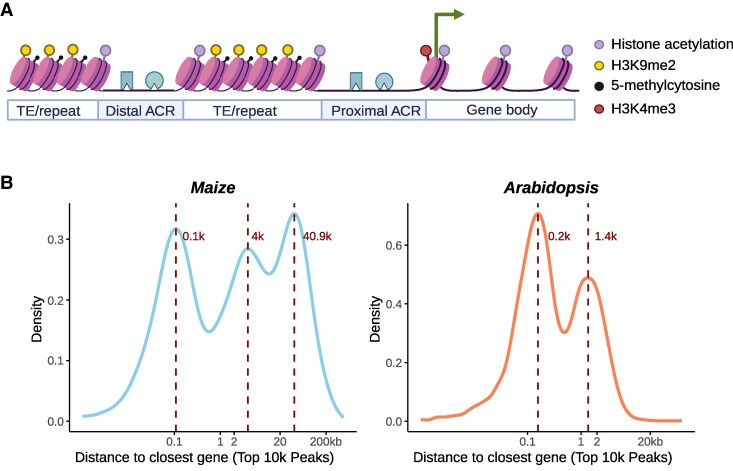
The presence of distal regulatory regions in large genomes. **A)** Schematic diagram generated by BioRender showing the nucleosome distribution of closed and accessible chromatin regions (ACRs) upstream of a gene. The ACRs are often nucleosome-free, with TF binding to cis-regulatory elements that can regulate gene expression. Histone acetylation such as H3K27ac and H3K9ac are often deposited at the nucleosomes flanking accessible chromatin regions as well as the transcribed gene body region. The first nucleosome near transcriptional start sites is marked with H3K4me3. The TE and repeat regions have condensed nucleosomes and are often associated with DNA hypermethylation and H3K9me2. **B)** Maize genes often have additional distal ACRs. The distribution of distance of the summit of ATAC-seq peak to the nearest gene's transcriptional start site is shown. To avoid the noise in cross-species comparison, only the top 10,000 peaks from Arabidopsis and maize leaf ATAC-seq based on signal fold change are used (Tu et al. 2022). Including the weaker ATAC-seq peaks will not change the pattern but will increase the height of maize 4-kb and Arabidopsis 1.4-kb peaks.

While Arabidopsis is an invaluable model for plant research, it does have certain limitations that we should keep in mind. It has a very compact genome, where the gene regulatory regions (transcription factor–bound and nucleosome-depleted accessible chromatin regions) are in close proximity to the genes they control. This differs from many crop species, where there is more genomic space between genes to evolve distal regulatory regions. If we plot the distance distribution of the ATAC-seq peaks to nearest genes, the Arabidopsis data shows 2 proximal accessible chromatin regions (ACRs) peaks within 2 kb of the genes’ 5′ putative promoter region (Fig. 6). In contrast, the 2-Gb maize genome has additional peaks at 4 and 40 kb, suggesting that distal regulatory regions are common for maize genes. Therefore, when studying gene expression, the common practice of cloning a 2-kb upstream “promoter region” and fusing it to a reporter gene would work for Arabidopsis without the need of considering the location of ACRs. But it would be less effective for large-genome crops, where critical regulatory elements can be located in ACRs much further away from the target genes.

The presence of distal regulatory regions in those large genome plants is particularly important for genome engineering. While some early studies using multiple guide RNAs (gRNAs) to randomly target the 5′ upstream regions of genes were able to alter gene expression (Rodríguez-Leal et al. 2017), we should consider that if the key cis-regulatory elements controlling the gene's expression are further upstream, none of those randomly selected gRNAs would hit the critical region harboring those regulatory sequences. Hence, it is better to first determine the position of potential ACRs that regulated the target gene via ATAC-seq or DNase-seq, or in some cases, examine Hi-C or ChIA-PET data to see whether there are distal regulatory regions that are further upstream, before designing genome editing strategies. An interesting and underexplored question is whether, when attempting to recreate a gene's expression pattern in a synthetic construct, it is necessary to clone the distal regulatory elements along with the intervening “gap” DNA sequence. Alternatively, could one simply fuse the distal regulatory elements directly to the 5′ end of the gene and still obtain the same expression pattern?

The extra genomic space between genes in crop plants is filled with transposable elements and other repeats that tend to be heavily methylated to prevent them from being expressed. It would not be surprising if plants have evolved mechanisms to leverage these epigenetic marks to create new gene expression patterns and traits. For example, during tomato fruit development, the genome undergoes a global demethylation, affecting both genes and transposable elements (Zhong et al. 2013). This coincides with the appearance of new ACRs in gene promoters, enabling key transcription factors like EIN3 and MADS-box RIN to bind to cis-regulatory elements and activate genes that were inaccessible in other tissues (Lü et al. 2018). Hence, one might need to consider tissue-specific ATAC-seq data to identify regulatory regions that might control the target gene expression. Profiling ACRs in certain tissues can sometimes be technically challenging. Since TEs and repeats in the plant genome must be silenced by DNA methylation, and ACRs are often unmethylated regardless of tissue specificity, it has been demonstrated that whole-genome methylation data can be utilized to identify unmethylated regions, thereby allowing for the localization of ACRs and cis-regulatory elements (Crisp et al. 2020). Additionally, H3K9me2 ChIP-seq data could serve as an alternative to DNA methylation data, while histone tail acetylation ChIP-seq such as H3K27ac and H3K9ac could be used to identify histones flanking the ACR (Fig. 6). It is also possible to conduct in silico sequence comparison using genome data alone to identify the conserved noncoding sequences between or within species, which have been shown to cover most of the ACR and cis-regulatory elements (Song et al. 2021).

Many nonmodel species are difficult to transform and have long generation times. This often leads researchers to express genes heterologously in model plants, which can produce misleading phenotypes and wrong functional inferences due to species-specific differences. For example, the ethylene-dependent fruit ripening process in tomato is absent in Arabidopsis. The tomato MADS-box transcription factor RIN can bind to ethylene biosynthesis genes ACS1/4 and ACO1, while ethylene can activate EIN3 TFs to upregulate RIN, forming a positive feedback loop that is crucial for the ripening process (Lü et al. 2018). Although Arabidopsis has orthologous ACS, ACO, MADS-box, and EIN3 genes, their promoters lack the relevant cis-regulatory elements to establish this regulatory circuit. Simply expressing the tomato TF or ethylene biosynthesis genes in Arabidopsis will not recapitulate the ripening phenotype, making it nearly impossible to use Arabidopsis to study unique biology processes in other crops.

This issue may also arise when studying “conserved” biological functions. The GOLDEN2-LIKE (GLK) transcription factor is considered a conserved regulator of chloroplast development and photosynthesis across plant species. Mutations in GLK often result in pale-green leaf phenotypes, and the GLK gene from one species can complement the GLK mutant in another plant (Wang et al. 2013). However, cross-species comparison of GLK ChIP-seq have shown that over 80% of GLK's binding target genes are species-specific (Tu et al. 2020, 2022; Yelina et al. 2024). But the few conserved GLK target genes are susceptible to the GLK loss-of-function mutation and most of them are related to photosynthesis; hence, we observed the same pale-green leaf phenotype in different species. It demonstrates how rapidly cis-regulatory elements in gene promoters can change during speciation. We should not assume that the regulatory relationships found in 1 species will necessarily translate to other species. It is important to note that most studies focus on how the promoter ACRs located in the 5′ upstream region can influence gene expression. A recent study comparing the tomato and Arabidopsis CLAVATA3 gene demonstrated that mutations in both the 5′ and 3′ ends of the gene can have a synergistic effect on their expression, with mechanisms differing between species (Ciren et al. 2024). This indicates that the evolution of the structure and position of regulatory elements in a conserved regulatory gene could also lead to phenotypic changes.

Despite many plants having evolved complex transcriptional regulations that are not present in Arabidopsis, the underlying molecular mechanisms of TFs, cis-regulatory elements, and epigenetic control over gene expression remain the same for all plants. Arabidopsis will continue to serve as an ideal model system to study those. However, researchers must be mindful that the changes in cistrome and epigenome can result in both within species and species-specific differences, even for biological processes that are highly conserved.

## Functional phenotyping of plant acclimation responses to drought stress: what constrains transfer from Arabidopsis to crops, and vice versa?

(Written by Francesca Cardinale, Ivan Visentin, and Claudio Lovisolo)

As the previous sections have made clear, there may be difficult aspects to tackle for the translation of molecular findings among species. At least as many can be run into when shifting to the phenotypic level—and this is even more true for stress-related findings, where external variables add complexity. A systematic comparison of stress responses requires first to identify the main research purpose and the most suitable species for achieving it. In this frame, there are 2 fundamental dimensions to the translation of stress-related findings from Arabidopsis to crops, and vice versa. If we phenotype for functional genetics, proxies of stress responses and performances by remote sensing are the best option: although rough/more indirect, they allow automation and upscaling. High-throughput phenotyping is certainly an asset more easily applied to Arabidopsis than other species, together with the wealth of genetic and molecular tools and the advantages of a short life cycle and size (Korwin Krukowski et al. 2020). However, if we phenotype to decrypt specific features of, for example, drought responses, a more nuanced physiological evaluation is needed, and Arabidopsis may not be the obvious choice. Also, application-driven research requires direct hypothesis testing on crop plants. There, things may become more complicated.

To state the obvious, not all plant species are genetically adapted to the same environment: what can be stressful to one species may not be for another. For example, differences in metabolism, anatomy, and overall morphology will change the time it takes for a plant to feel the strain from a drought episode, altering water relations among organs and reactions to stress as a consequence. However, once conditions become suboptimal, all will at least attempt to acclimate. Most species—including Arabidopsis—will have at their disposal a mix of strategies to cope with stress: avoidance (especially via stomatal closure), tolerance (especially via osmoregulation and antioxidant defenses), and/or escape (via a change in the duration of the developmental cycle; not discussed here). What strategy, or mix of strategies, different species may adopt in any given situation is thus first and foremost linked to their evolutionary adaptation to low water availability. However, other conditions—for example, soil type, stress intensity and speed, history of previous stress episodes—may shift the choice of strategy in individual plants in favor of avoidance vs tolerance (Gilbert and Medina 2016). Such flexibility may also change the causal order of individual strategies among species. For example, the osmoprotective responses typical of low water potential (ψ_w_, an index of water availability) can occur as a result of environmental shortages of available water or, on the contrary, can be the consequence of transpirative dissipation. The latter happens in plants with inefficient stomatal control that keep transpiring profusely despite incipient stress conditions. Even the triggers for the biosynthesis of antioxidant compounds, which are always linked to imbalances between the radiant energy absorbed by the leaves and its subsequent use in ATP synthesis and chloroplastic reducing energy, can vary in their timing and thus be more or less responsible for tolerance in different species. Again, there may be an intrinsic lack of water or instead a dysregulation of its use, and acclimation responses will follow the kinetics of balancing between leaf ψ_w_ and transpiration losses.

**Figure 7.**
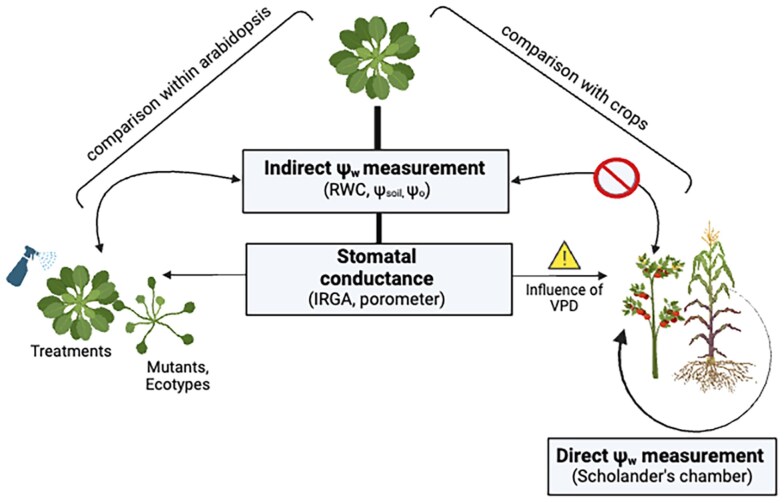
Water potential (ψ_w_) is the variable most directly describing water status. The description of acclimation responses is only meaningful within water potential values between the first signs of stress and nonreturn values; however, this range varies in different plant species and cannot be transferred without wet lab/experimental confirmation. ψ_w_ can be directly quantified in most plants by a Scholander's chamber, but this is not so straightforward in Arabidopsis. Thus, other, more indirect variables are normally measured, such as relative water content (RWC), water potential in soil (ψ_soil_) or osmotic potential in tissues (ψ_o_). Plant acclimation strategies to water status variations during both water deprivation and rehydration routines modulate stomatal conductance (g_s_) and transpiration, measured by various means. However, the relationship between these variables and ψ_w_ (and its indirect proxies) is not the same in Arabidopsis and crops, which once again complicates the transfer of meaningful information; in addition, stomatal conductance will be affected by vapor pressure deficit (VPD) between leaf tissues and air, which can vary dramatically for semi-controlled environments and field experiments. Although a thorough characterization is missing, the g_s_/ψ_w_ relationship in Arabidopsis seems to follow an intermediate curve between the one describing tolerance vs avoidance strategies.

So, first, when setting up an experiment to investigate the dynamics, extent, and nature of acclimation mechanisms in your species of interest, you need to know your plant's water status (Juenger and Verslues 2023) and the ψ_w_ range in which, in your experimental conditions, it will start experiencing stress vs reaching a point of no return; you must work between these boundaries. In fact, acclimation is not needed for less negative ψ_w_ values, while at more negative ones it will be useless (Fig. 7). While the ψ_w_ of the plant is brought down into such range in a controlled way, you will be able to make sense of the physiological changes being observed, in light of the different acclimation strategies possibly adopted by your plant species (e.g. osmoprotection vs stomatal closure vs changes in the antioxidant status). In fact, the curve describing the relationship between stomatal conductance (g_s_) and ψ_w_ is generally quite informative on the kind of acclimation strategy the plant is adopting also in response to different soils or treatments. A very extended curve along g_s_ but rather narrow in ψ_w_ variations describes a drought stress avoidance strategy, probably linked to signaling from the root towards the stomata (Visentin et al. 2016, 2020). On the contrary, a broad range of variation in the ψ_w_ field means that a stress tolerance strategy is prevailing and that the plant is not heavily relying on the control of transpiration but rather putting forward strong lines of defense against evaporative water loss (osmoprotection, ROS scavenging, control of xylem embolization, etc.). Also note that sharp bends in the shape of the curve may indicate a response threshold on which to focus for investigating the molecular underpinnings of acclimation and may also suggest thresholds for intervention (e.g. irrigation in crops). Following the ψ_w_ vs g_s_ dynamics will also allow you to lower if not to zero out confounding factors such as different canopy sizes in mutants of the same species.

What becomes crucial then, is to find the boundaries of this “acclimation space” by reliably measuring the leaf ψ_w_. However, in this respect, Arabidopsis is far from being the most convenient model. In most plant species, ψ_w_ and related physiological parameters can be measured in the leaves with modest investment in equipment and effort. For them, there is a trove of reports on leaf ψ_w_, often taken at midday or with day-long kinetics; on ψ_w_ within the shoot, by the proxy of a bagged leaf; and on pre-dawn ψ_w_ as a proxy of ψ_soil_. Unfortunately, the Scholander's chamber, the gold standard to the purpose, is used with difficulty on Arabidopsis due to 3 main morphological features: few leaves, bunched together in a basal rosette, and equipped with a relatively short petiole. Sacrificing 1 or more leaves at each measurement significantly lowers the transpiring surface; this means that the first and subsequent measurements cannot be reliably compared during desiccation and rehydration kinetics. Multiple leaf sampling also alters the light regime of the remaining leaves in the basal rosette: this again may change transpiration and assimilation rates compared with the pre-measurement condition. Finally, the ideal petiole should be long enough for a robust pressure seal gasket to withhold a few tens of bars of compressed air in the Scholander chamber; it is not so in Arabidopsis (Rodriguez-Dominguez et al. 2022). To overcome such issues, the whole epigean part can be inserted into the chamber. In this case, however, the measured ψ_w_ is from the shoot and thus significantly less negative than the standard leaf ψ_w_ that is mostly reported from other species. Additionally, since each measurement kills a plant, the number of replicate individuals needed for a full desiccation/rehydration time course increases considerably. The above reasons have pushed plant scientists to resort to more indirect measures, that is, proxies of leaf ψ_w_ that we will briefly summarize below.

Tissue osmotic potential (ψ_o_) can be estimated on ground material or leaf disks with a precision thermocouple transducer in a chamber functioning either as a psychrometer or as a hygrometer (wet bulb vs dew point depression method, respectively). Also, relative water content (RWC) in leaves is often assumed to be proportional to the leaf ψ_w_ (Verslues and Bray 2004). The same kind of assumption is behind the measurement of soil water potential (ψ_soil_) with tensiometers or psychrometers; or the transfer to agar substrates with known PEG content, which should both correlate with ψ_w_ in the roots (van der Weele et al. 2000). This assumption, while useful to produce and compare findings in Arabidopsis, cannot be translated quantitatively to other species, since the relation between RWC and ψ_w_ or ψ_soil_ can vary enormously in different biological models. Also, this relatively broad range of experimental options—barely including direct ψ_w_ measures—has unsurprisingly led to widely variable published estimates of ψ_w_ at which Arabidopsis can be considered stressed but still able to recover.

Contrarily to ψ_w_, g_s_ in Arabidopsis is not difficult to quantify via gas exchange measurements by infra-red gas analyzers (IRGAs) or porometers (Ceciliato et al. 2019) but is influenced by air vapor pressure deficit (VPD) between air within and outside the leaf (Busch et al. 2024). For this reason, stable environmental conditions during measurements become crucial but are nearly impossible to achieve in the greenhouse and difficult in growth cabinets with little air replacement or in whole-plant chambers (Patono et al. 2022, 2023). In the event that transpiration is determined with IRGAs, an added value to the measurement of water vapor exchange between the plant and the atmosphere is given by the parallel measurement of CO_2_ exchange. Keeping track of the timing and extent of transpiration and carbon assimilation or, on the contrary, of (photo)respiration is imperative to attribute stomatal control and water loss vs conservation to metabolic causes. Alternatively, transpiration (and thus, gravimetric methods that weigh plants and pots continuously, as in phenotyping platforms) is a good proxy for g_s_ if VPD is stable or varies in a controlled way for all measures and is recorded as air temperature and relative humidity throughout the experiment.

What is the g_s_/ψ_w_ relationship in Arabidopsis, then? Given the mentioned instrumental difficulties in direct quantification, ψ_w_ in Arabidopsis has not been thoroughly studied yet under a range of experimental conditions. However, g_s_/ψ_w_ values seem to follow neither strictly the typical stress avoidance nor the stress tolerance curve in this species, as the few reported ones generally lie in between (Thonglim et al. 2023). This intermediate behavior is simultaneously a limit, as it does not allow to investigate either strategy alone, and an advantage, as it approximates both. Finally, it is necessary to consider here another major limitation to translation of stress-related molecular findings from Arabidopsis to crops. In the latter, the strength of sinks (roots and especially fruits) is the result of centuries of domestication and decades of breeding. Furthermore, crop engineering is increasingly focusing on optimizing phloem downloading by refining invertase activity. Such strategy can accelerate agronomical yield in irrigated crops under rising temperatures, a result of the current climate crisis (Lou et al. 2025). Stronger sugar downloading triggers faster carbon assimilation, strictly coupling stomatal opening and closing to metabolism, sensed as residual CO_2_ concentration in the leaf intercellular spaces; but it exacerbates water conservation problems by the plant. The transpiration control system of crops is therefore predicted to become more and more difficult to translate to Arabidopsis (and vice versa), as stomatal control under stress in the latter is fundamentally based on the ABA signal and the ψ_w_ of the root/leaf system. To add further complexity, stomata are also controlled by different mechanisms at different ambient CO_2_ and VPD levels, another key shifting variable linked to global warming (Koolmeister et al. 2024).

Thus, we support the call for the adoption of ψ_w_ as a fundamental descriptor of plant water status, that can enhance the insights gained from many drought-related experiments and facilitate data integration and sharing (Juenger and Verslues 2023). We highly recommend following also its interrelationship with the g_s_ variation range but recognize the actual limitations in applying these recommendations to Arabidopsis (Thonglim et al. 2023).

To achieve maximum mutual benefit, the Arabidopsis community must do their best to describe their drought response experiments not only with time courses but at least reporting detailed variations in leaf RWC, possibly along with g_s_ and ψ_w_. Conversely, the crop physiologist who draws on Arabidopsis molecular databases, or on the results of comparisons among mutants, must document to what extent the g_s_/ψ_w_ response model of Arabidopsis copies that of the species under study, and transfer with care the available data to their own biological system.

## Arabidopsis research provides an essential but incomplete framework for translational research in the field of circadian biology

(Written by Matthew A. Hannah, Alex A.R. Webb)

### Arabidopsis research has provided a detailed model of a plant circadian system

It was known that plant physiology is modulated by circadian clocks centuries before Arabidopsis caught plant scientists’ attention. Studying *Phaseolus* leaf movements in the 1930s, Bünning discovered aspects such as temperature compensation and inheritance and developed conceptual models for flowering time regulation. The following decades saw known circadian-regulated processes extended to include growth, stomatal movement, and germination and to molecular analyses in the mid-1980s (see McClung 2006). Transcriptional rhythms were first demonstrated in pea and other crops but soon included Arabidopsis (Millar and Kay 1991). This early labor intensive work rapidly accelerated, benefitting from new Arabidopsis tools including genome sequence, mutation collections, and efficient transformation.

Arguably the most impactful tools were circadian-regulated gene promoters driving luciferase reporters (Millar et al. 1992). This enabled forward genetic screens to identify numerous circadian *loci*. Combined with other tools, and a notably early adoption of mathematical modeling (Locke et al. 2005), this allowed the identification of the circadian oscillator genes that underlie rhythm generation, entrainment mechanisms, and other aspects of rhythmic biology for which the circadian oscillator acts as a timekeeper, such as flowering time (Creux and Harmer 2019; Wang et al. 2022a, 2022b), photosynthesis, and growth (Dodd et al. 2005). The Arabidopsis model has provided a conceptual framework on which hypotheses about circadian gene function can be made—a huge resource, unlikely to be reproduced in the near future in crop species.

**Figure 8.**
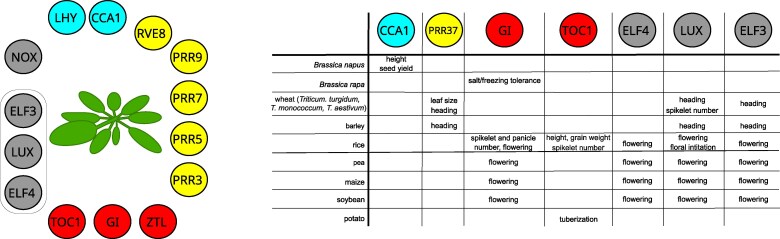
Identification of circadian oscillator components has informed analysis of the effect of circadian *loci* on phenotypes in crops. **A)** Arabidopsis circadian oscillator components ordered by sequence of approximate activity where blue indicates dawn (LHY to CCA1), yellow the day (REV8 to PRR3), red dusk (ZTL to TOC1), and grey the night (ELF4 to NOX). CCA1 (CIRCADIAN COLD ASSOCIATED 1), LHY (LATE ELONGATED HYPOCOTYL), RVE8 (REVEILLE 8), PRR (PSEUDO RESPONSE REGULATOR), ZTL (ZEITLUPE), TOC1 (TIMING OF CAB EXPRESSION1), GI (GIGANTEA), ELF (EARLY FLOWERING), LUX (LUX ARRHYTHMO), and NOX (NOX/BROTHER OF LUX ARRHYTHMO). The box around ELF3/4 and LUX indicates they work as an evening complex of proteins in Arabidopsis. **B)** Some effects of orthologues of Arabidopsis circadian oscillator components on yield-related phenotypes in crops. See Supplementary Table S1 from Steed et al. 2021 for references.

Insight from Arabidopsis accelerated discovery of circadian-associated *loci* in crops that facilitate adaptation to local environments or provide advantageous traits (Fig. 8; Steed et al. 2021). This includes identification of causal gene/mutation from a candidate list or using Arabidopsis mutants for complementation to find conserved, or partially conserved, function of crop genes (Supplementary Table S1 in Steed et al. 2021). However, it cannot always be directly translated to crop systems. *ELF3*, *LUX*, and *GI* are involved in the regulation of flowering time in wheat and Arabidopsis. Based on Arabidopsis it might be assumed that their effects on heading (flowering) arise due to changes in the circadian timing of the peak of the coincidence detector CO (Suárez-López et al. 2001). However, the wheat photoperiodic network is different to Arabidopsis (Alvarez et al. 2023); CO is not a major regulator of flowering, and ELF3 can affect flowering independently of its role in circadian oscillators (Wittern et al. 2023). Orthologues of the Arabidopsis circadian oscillator components *PRR3* or *7* are major regulators of flowering in barley and wheat but have no known role in the circadian oscillators of these cereals (Turner et al. 2005). These examples show that while the framework is conserved, the nuances of specific roles may be lost if crop studies are not included.

### The ins and outs of circadian regulation are less understood than oscillator structure

Detangling the pleiotropic effects of circadian regulation to understand how oscillator components regulate individual biological pathways benefits from Arabidopsis resources. It remains a goal to identify the pivotal changes that result in 30% of the transcriptome being circadian-regulated and which of these changes specifically are causal for altered biological activity (Harmer et al. 2000). Detailed analysis of the binding and regulatory activity of Arabidopsis transcription factors, including circadian oscillator genes and the transcriptional cascades they initiate, is essential for understanding how circadian oscillators regulate specific pathways. For example, the identification of TOC1-bound regulatory promoter sequences in Arabidopsis (Gendron et al. 2012) has allowed us to suggest a role for wheat TOC1 in phasing the timing of *ELF3* to dawn to regulate heading (Wittern et al. 2023). The progress on diel- and circadian-regulation of transcription needs to be extended to protein abundance/activity because there is a poor correlation to protein abundance (Graf et al. 2017). Here, Arabidopsis will help overcome technological challenges of quantitative proteomics and better link to biological pathways by allowing correlations with other -omics data (Scandola et al. 2022). Protein structural biology will be important to understand the temporal regulation of protein-protein interactions, phase separation, condensation, and nuclear-cytosolic translocations that underpin circadian regulation of outputs (Creux and Harmer 2019). Arabidopsis genetic variation and analysis of resultant phenotypes has been powerful in interpreting how variation in the liquid-liquid phase separation of ELF3 in vitro is related to its role as a thermosensor (Hutin et al. 2023). Extension of protein studies to other circadian genes will inform how they regulate signaling, metabolism and development may solve how the oscillating abundance of clock transcription factors regulate physiological pathways, such as stomatal movements, that are not yet fully understood. With many gaps remaining, Arabidopsis research will continue to be important to increase our knowledge of circadian input and output pathways. It will be important to also utilize additional model and crop species to avoid neglecting the many pathways that are circadian regulated but not well-represented by Arabidopsis, such as specific disease responses, secondary metabolic pathways, or metabolism in specialized tissues or organs.

### Important targets for future translational Arabidopsis research

Since the circadian clock is a master regulator, biotechnological approaches might benefit from specific mechanistic insights identified in Arabidopsis that uncouple desired traits from undesired pleiotropic effects. One area where such mechanistic insight is required, that might deliver future yield benefits, is carbon cycling and allocation. All plant species appear to store a carbon reserve during the day, that is mobilized at night to support respiration and growth. In Arabidopsis this is starch (Lu et al. 2005); in cereals sucrose is also important (Zepeda et al. 2023). Recently, it has been discovered that the ability of Arabidopsis to turn light energy into biomass is greater in the second half of the photoperiod (ZT8-16 h), than the first (ZT0-8 h) (Wang et al. 2024). How these processes are regulated is not known, but studies in Arabidopsis which have both implicated and excluded circadian oscillator components (Alexandre Moraes et al. 2022) will continue to be important. The contribution of circadian oscillators to biomass accumulation, and particularly the mechanisms by which this occur, have primarily been studied in Arabidopsis. However, this focus does not capture the circadian regulation of photosynthesis and metabolism in C4 and CAM plants. Comparisons between C3, CAM, and C4 might be very powerful in identifying key regulatory control mechanisms because of the different phasing of activities between night and day.

Arabidopsis can contribute to enable faster and more extensive testing of new concepts around the relationships between the internal rhythms and those of the external environment. Ecological studies are establishing how circadian systems contribute to plant fitness in the “real world” (Hubbard et al. 2018) and how circadian oscillators respond to changing light in twilight (Mehta et al. 2024). Since most of the circadian research has taken place in controlled environments using constant and square wave cycles of temperature and/or light, additional studies will be essential to understand the biology needed to improve outdoor crops in a changing environment. Arabidopsis research could also help understand how best to optimize the genotypes and growth conditions in the expanding field of controlled environment farming, such as in vertical farms. This is a particularly exciting field since being able to fully control the environment expands the potential for phenotypic variation and may enable improved productivity and reduce environmental impacts through the translation of fundamental circadian biology research to farm application, a process we have termed “chronoculture” (Steed et al. 2021). However, considering the diversity of crop species, environmental conditions, and agricultural management systems, further crop research will also be crucial if such insights are to be effectively translated into agricultural practice.

## Data Availability

There are no new data associated with this article.

## Dive Curated Terms

The following phenotypic, genotypic, and functional terms are of significance to the work described in this paper:

EIN3 Gramene: AT3G20770
EIN3 Araport: AT3G20770

GNC Gramene: AT5G56860
GNC Araport: AT5G56860

SHR Gramene: AT4G37650
SHR Araport: AT4G37650

crc Gramene: AT1G69180
crc Araport: AT1G69180

MYB36 Gramene: AT5G57620
MYB36 Araport: AT5G57620

RIN Gramene: Solyc05g012020
RIN Araport: Solyc05g012020

PPDK Gramene: AT4G15530
PPDK Araport: AT4G15530

ACO1 Gramene: Solyc07g052350
ACO1 Araport: Solyc07g052350

ELF3 Gramene: AT2G25930
ELF3 Araport: AT2G25930

PEPC Gramene: Sobic.010G160700
PEPC Araport: Sobic.010G160700

TOC1 Gramene: AT5G61380
TOC1 Araport: AT5G61380

PEPCK Gramene: Sobic.001G432800
PEPCK Araport: Sobic.001G432800

aerenchyma AmiGo: PO:0005702

LHY Gramene: AT1G01060
LHY Araport: AT1G01060

RWC Gramene: Relative Water Content
RWC Araport: Relative Water Content

Gigantea Gramene: BRADI2G05226
Gigantea Araport: BRADI2G05226
